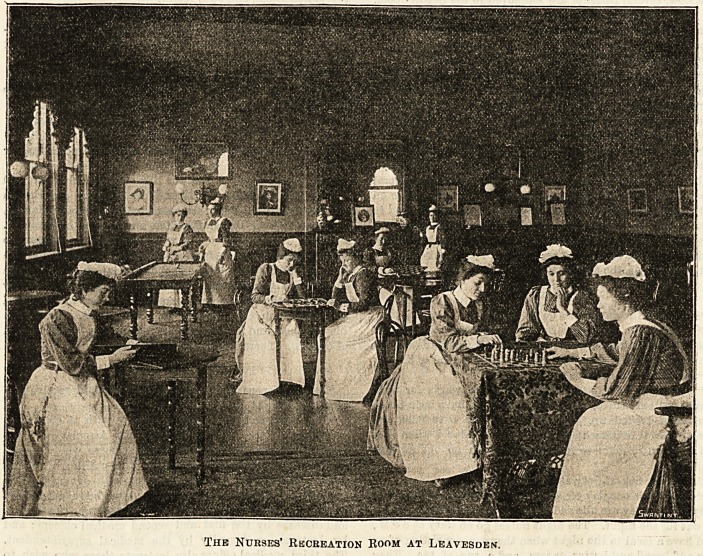# Nursing Section

**Published:** 1904-02-20

**Authors:** 


### The Hospital.

**Nursing Section.**

Contributions for this Section of "The Hospital" should be addressed to the Editor, "The Hospital" Nursing Section, 28 & 29 Southampton Street, Strand, London, W.C.

No. 908.—Vol. XXXV. *SATURDAY, FEBRUARY* 20, 1904.

### Notes on News from the Nursing World.

### QUEEN ALEXANDRA'S MILITARY NURSING SERVICE.

We are officially informed that Miss S. Smyth and Miss G. Knowles have been provisionally appointed staff nurses in Queen Alexandra's Imperial Military Nursing Service; also that Sisters J. E. Dods and and H. Suart, together with Staff-Nurses K. Pearse, E. H Hay, A. R. F. Auchmuty, and D. M. Taylor, are confirmed in their appointments, their period of probational service having expired.

### THE QUEEN'S COMMEMORATION FUND.

A very wise step was decided upon at the annual meeting of the Queen's Commemoration Fund last week. In order to maintain, or, as we prefer to put it, to strengthen, the representative character of the Committee, it was resolved to invite the Roman Catholic Archbishop of Westminster, the President of the Wesleyan Conference, Dr. Clifford, and Lady Dimsdale, to join the executive. Lady Dimsdale of course, has been asked because of the valuable work she did in respect to the women's memorial to Queen Victoria. But the other invitations give emphasis to the fact that the Fund is, as it should be, administered without regard to religious differences. Last year the amount collected on behalf of Queen Victoria's Jubilee Institute for Nurses, in subscriptions and donations, was 3,733*l.* We hope that this year, with a stronger Committee, the sum will be considerably larger.

### THE RUSSIAN SICK AND WOUNDED.

With regard to the arrangements for nursing sick and wounded Russian soldiers during the war with Japan, we are officially informed that the war was such an entire surprise to the authorities that the Russian Embassy in London has no information on the question. Any English nurses, therefore, who desire to volunteer for service should write direct to the managers of the Red Cross Society, St. Petersburg. The Russian Ambassador thoroughly appreciates the desires which have been expressed to afford help, and in the meantime it may be pointed out that in the Russian capital the Dowager Czarina—who, it will be remembered, is sister to Queen Alexandra—has opened a branch of the Red Cross in the New Hermitage of the Winter Palace.

### NO FOREIGN NURSES FOR JAPAN.

Notwithstanding the statement we made before the war between Japan and Russia broke out, that the aid of foreign nurses would not be required by the Japanese Government, we have since been asked by several English nurses if in the event of their services being volunteered, there would be any chance of the offer being accepted. We have, therefore, made inquiries on their behalf at the Japanese Legation, and the Minister, Viscount Hayashi, whilst expressing his high appreciation of the kind proposal, tells us that although he has not yet had any direct communication on the subject from the Government at Tokio, he believes that the Japanese army nurses themselves will amply suffice for all needs. His Excellency also says that, however well a nurse may have been trained, inability to speak the Japanese language is a serious drawback. For these reasons, the offer which has again been made to send a hundred American nurses to the seat of the war, will doubtless be declined. But if any English nurses desire a further assurance, they should address their communications to the Red Cross Society, Tokio, as no other answer can be given by the Legation in London.

### LORD DUDLEY AND ST. PATRICK'S NURSES.

At the annual meeting of St. Patrick's Nurses' Home last week, presided over by the Archbishop of Dublin, the Lord Lieutenant of Ireland showed his interest in the institution by attending and delivering a short speech. Lord Dudley warmly commended the work done by the nurses amongst the poor of Dublin and in remote districts in the West of Ireland. His Excellency said that he had seen a little of the work of nurses himself, and he was convinced that no greater boon could be provided for the ordinary lives of the working classes than the provision of trained nurses for the sick. His commendation is abundantly justified by the report of the home, which shows that during the past year the number of cases nursed was 2,000, and the number of visits paid 37,000.

### PARLIAMENT AND STATE REGISTRATION.

It has been suggested that the progress of the Bill for the State Registration of Nurses will be "blocked" by members who desire to prevent it from becoming law. But there is really no cause for resort to the tactics suggested. The Bill in charge of Dr. Farquharson has not the remotest prospect of being debated this Session, its promoters having failed to obtain a place on the paper which would give it a chance of consideration. In any case, the nurses who, not having been trained for three years, write to ask us how they will be affected by legislation, need not be concerned; they are not likely to be interfered with so long as they do their duty.

### THE STANDARD OF MENTAL NURSING.

It will be observed from the report of the interview of our Commissioner with the matron of Leavesden Asylum that Miss Howell is a keen supporter of a high standard of mental nursing. She claims that it ought to come before any other kind of nursing, and as she is herself a hospital-trained, as well as an asylum-trained nurse of considerable experience, her opinion is entitled to consideration. It will probably, however, be maintained that the cure of persons who are mentally afflicted being much less frequent than that of those who suffer from diseases of the body, the nursing of the latter is of more importance to the world; and the argument is one which cannot be lightly set aside. As to the nursing of the mental sufferers being more difficult, we should rather be inclined to say that it is more wearing and thankless. We certainly welcome the enthusiasm which the matron of Leavesden manifests in her responsible and onerous position. In insisting upon the need of the best possible training for mental nurses, she strikes the right note, and we are glad to learn that the conditions of work on the part of the staff under her control are shortly to be improved by the opening of a nurses' home of the same complete description as the homes attached to modern general hospitals and Poor-law infirmaries.

### DISTRICT TRAINING AT KEIGHLEY HOSPITAL.

With reference to our notice of the fact that the superintendents of some of the principal hospitals in Chicago have decided that in future probationers in their training schools are to have a month of district or visiting work added to their curriculum, we learn that for some time past it has been the custom for the senior probationers at Keighley Hospital to take turn as "district probationer." In fact, the regulations drawn up for intending probationers contain a clause to the effect that "during their third year probationers may be appointed staff nurses in a ward, or they may be sent out to nurse private cases, or to act as district nurses." In either of the latter events they receive extra pay. There is a fully-trained district nurse, maintained by the Charity Organisation Society, who lives in the hospital, but the work is more than one nurse can manage, and, accordingly, a probationer goes out to help her every morning. The new matron, Miss E. C. Laurence, now proposes to give each probationer in her third year two months' district work. This, the completion of the new Nursing Home—into which the nurses moved a few days ago—and the enlargement of the Hospital, hereafter to be called the Victoria, will doubtless add considerably to the popularity and usefulness of the training school at Keighley.

### ONE NURSE FOR 98 CASES.

At the last meeting of the Cannock Guardians the workhouse master reported that about 2 A.M. on January 18th a delirious patient climbed out of a window of the infirmary. He was missed, and found a short time afterwards on a heap of dirty clothes on to which he had fallen, a distance of 12 feet. If his fall had not been broken he would probably have been killed, but as it was "he only," the report says, "bruised himself." Lord Hatherton, having asked the master how many nurses were available, he replied that "there was only one night nurse to look after 98 cases. There were six wardsmen, but these were old infirmary patients." The master went on to state that there were only two windows out of which persons could get, and "these had now been barred." Temporarily, additional help had been obtained, but the master added that "it was impossible for one nurse to watch 98 persons, and it would be for the guardians to consider what extra permanent help should be given." In the meantime, we may point out that if the delirious patient who climbed out of the window of the infirmary had been killed, the Cannock Guardians would have exposed themselves to severe criticism at least.

### REPORTED DISMISSALS AT TOOTING BEC ASYLUM.

We hear that eight female nurses at Tooting Bec Asylum for Imbeciles have been summarily dismissed for playing a practical joke upon a relief nurse whom they accused of making certain statements to the medical superintendent. Grievances are alleged on both sides, and it appears that the dismissed nurses contend that the punishment inflicted was unnecessarily severe. We may have something to say on the question next week.

### "IN THE GARB OF A PROFESSIONAL NURSE."

Whatever may be the result of the charge preferred against Marie Edwards, a woman who appeared at North London Police Court on Saturday in the garb of a professional nurse to answer charges of obtaining money by false pretences, it is certain that her assertion to a Dalston lady, from whom she obtained money, that she was a student nurse at the London Hospital, is not true. The other story, that she was a nurse at Netley Hospital, is, we are officially informed, equally devoid of foundation. Marie Edwards, the lady superintendent states, "has never been an Army nurse at Netley." But why did not the persons to whom she made such statements take the trouble to ascertain if they were accurate? Neither the garb of a professional nurse, nor any other garb, justifies failure to observe the ordinary precautions which are necessary when strangers ask people for loans.

### THE MEMORIAL HOME AT TUNBRIDGE WELLS.

The new home of the District Nursing Association at Tunbridge Wells has just been formally opened. It is 11 years since the organisation started work and it has grown steadily. Last September the nurses removed to Holly Lodge, which was purchased as a memorial to Queen Victoria, and is to be a permanent home. It is in a central position, and the interior is comfortable and refined. There are four nurses on the staff, but the building is roomy and will accommodate several more nurses as the need for them arises. One interesting feature about the acquisition of the new home is that it has been partly paid for by subscriptions collected by means of the "shilling collection cards," from the people who are so greatly benefited by district nursing, and who have been very enthusiastic—the poor of the town. At the annual meeting of the Association in the Town Hall recently, several addresses were given, and the title-deeds of the new house were then handed to Lady Henry Nevill, who takes a great interest in the work. After she had expressed her pleasure at being present, Lady Henry and other ladies drove to the home and handed the key to the superintendent

### A NURSE AND HER NOTICE.

The difficulty of inducing nurses to remain for any length of time in Poor-law institutions has agitated many Boards of Guardians, but they do not often solve it. Some have tried increasing the salaries and raising the standard of comfort, with varying degrees of success. At their last meeting, the Exeter Corporation of the Poor received the resignation of an assistant nurse who was appointed last October. She assigned no reason for leaving, but one of the members of the Corporation expressed the opinion that there must be something radically wrong, as nurses were continually leaving. There was no information given to bear out the view that there was anything "radically wrong," and, as the Governor observed, the nurse might have a thousand reasons for leaving. Subsequently, one of the members said that they did wrong in electing her, and another described her as "a moving plant." We do not think speculations of this kind will help the Guardians to obtain applications for vacancies. A nurse has a right to resign a post in a workhouse infirmary if she chooses without assigning reasons, and if she does not assign them, it is neither good policy, nor good taste, to suggest them.

### A NEW MOVEMENT IN WILTSHIRE.

The Duchess of Somerset has initiated a movement in Wiltshire for the establishment of a county nursing association in connection with Queen Victoria's Jubilee Institute. The Duchess herself will act as president, and it is stated that the objects of the association are to systematise and improve the quality of district nursing throughout the county, under expert supervision, to help districts to start nurses, to arrange for the training and supply of nurses, to supply emergency nurses when required, and to provide lectures on nursing and hygiene, where desired, by local committees throughout the county. The county superintendent will be a Queen's nurse, with a salary of £50 a year. The development of the movement will be watched with interest.

### THE HOLT-OCKLEY NURSES.

In order to assist the funds of the Benefit Nursing Association which provides nurses for the sick poor in villages on the Holt-Ockley system variety entertainments were given at Wokingham on Monday and Tuesday. We hope that a satisfactory amount was realised, and in any case the organisation is indebted to Mrs. Walter, of Bear Wood, for the efforts which she made in order to secure a great success. In her interesting account of the movement, which last month came of age, Mrs. Walter speaks of the nurses receiving a thorough training. We believe that many of them do excellent work according to their lights, but the training which they are given at a cottage or maternity hospital "for periods varying from six to twelve months," is not accurately described as thorough. Nor do we agree with Mrs. Walter that they are better suited than hospital or district-trained nurses to visit the homes of the poor. The real argument in favour of employing them is that they are less expensive, but if this view had been allowed to prevail in respect to nursing generally we should still have Sairey Gamps abounding.

### ANOTHER DISTRICT NURSE WANTED AT SWANSEA.

Although Swansea was one of the first towns in the country to adopt the district nursing movement, Sir John Llewelyn had to complain in his speech at the annual meeting of the Swansea and District Nursing Association that, after 20 years' work, it does not obtain the support which it ought to receive. An additional nurse is urgently required at St. Thomas, but as the deposit account of the association had to be drawn upon last year, the committee do not feel that they are in a position to engage one. The proceeds of a café chantant amounted to £33, but such sources of income as this, though always welcome, cannot of course be counted upon. A personal appeal to all classes, particularly the working classes, was advocated, and we have no doubt that this is the best advice which could have been given.

### PROMPTITUDE IN DISTRICT NURSING.

At the annual meeting of the Birkenhead District Nursing Society, the Rev. A. Hamilton King mentioned that a case deserving immediate attention occurred in his parish. He wrote to the matron of the institution by the eight o'clock evening post, and the very best reply that could have been received was provided when a nurse came to the house immediately following the delivery of the letter. This, he thought, was an example of promptitude worthy of recognition. It is by promptitude in their attention to cases that district nursing organisations do much to justify their existence. There is nothing that the sick-poor and their friends more resent than delay when the services of the nurse are sought, and though in some instances it is unavoidable, the Birkenhead Society, with its staff of a matron and six nurses, is probably, generally speaking, in a position to send help at once where it is clearly needed. We are pleased to observe that the financial position of the society is satisfactory, a feature of the year's income being the receipt of £62 in donations and fees from the patients.

### NURSES' WEDDINGS.

Nurse Helen Ferrer was married to Mr. W. Wall, on Saturday last, at All Souls' Church, Willesden, by the vicar. The nurses from the Abbey Nursing Institute opposite — to which staff she has belonged for some years—sat in their blue uniform in the front rows and an old nurse friend was bride-attendant. The matron came into church with the bride and gave her away. The wedding breakfast was given afterwards in the Nursing Home, and bride and bridegroom went away at 4 p.m. amidst showers of confetti. The bride wore her travelling dress of blue hopsack and white satin blouse, with a blue hat. She had many pretty presents from old friends and patients; the matron and nurses gave her a spirit lamp and kettle for the table. Last week also, at St. John's Ealing, Jessie Etna Mount, eldest daughter of Mr. W. Albert Mount, of Ealing, was married to Henry William Buckle of the Royal Engineers. The bride is a member of the Army Nursing Service Reserve and both she and the bridegroom served in South Africa during the war.

### The Nursing Outlook.

" **From magnanimity, all fear above;**
**From nobler recompense, above applause,**
**Which owes to man's short outlook all its charm.**"

### THE NURSES' CO-OPERATION.

THE thirteenth annual report of the first Co-operation of Trained Nurses reminds us strongly how recent is the growth of organisation and the development of independence in the nursing world. The thought is consoling, for the report contains many old evils we had expected to see eliminated; but perhaps we have demanded too much in the way of business aptitude and strength of mind from those whose cause we have tried to champion. Time must be given to nurses, as to all women, to combat the servitude due to old habits and customs. It is so natural to a nurse to be scolded and bullied and ordered about and ignored that when those she has herself chosen to manage her business take up this tone she meekly submits. Therefore it is that the nurses of the Co-operation again met on one day and the members on another; that there was no public meeting where press and friends could attend and all the doubtful points which have caused so much friction for so many years could be debated openly, and so, probably, satisfactorily settled. And though the nurses at their own meeting were keen to have some of the year's balance applied to sick pay or annuities, it was the members' meeting which decided that the money should go to the reduction of the percentage. Is it right that the money earned by the nurses should thus be disposed of at a meeting attended by six persons only, none of whom are co operation nurses? The members present were Dr. Turney, Dr. Wallace, Miss Clutton, Mr. Bickersteth, Miss Leigh, Miss Morten; and though their aims and methods are above suspicion, in principle the government of the co-operation should be in the hands of its workers. It was one of the original objects of the association—

To establish and hold in trust funds for the provision of sick pay, pensions, or annuities for, or otherwise for the benefit of nurses; or pay any of such funds, to the National Pension Fund for Nurses, or the objects thereof, or combine in any manner with or assist the National Pension Fund for Nurses.

and as the older nurses who joined the Co-operation from 10 to 13 years ago find their powers failing and old age approaching it will become a very serious question for them. And in due course it will become a very serious question even for those who joined only last year. It is true that the Co operation nurses earn excellent salaries, and in a proper spirit of independence could easily each make provision for her own future. But such spirit is no longer fostered at 8 New Cavendish Street, and therefore cannot be relied on.

Once again we must call attention to the rule which for the first three or four years appeared in each report of the society—" Any surplus remaining shall be available for distribution as may in general meeting be determined"—a rule which has been quietly dropped since the present clique came into power, and nowadays it seems impossible to ever secure a "general meeting." Miss Kimber, late assistant superintendent of nurses of New York City Hospital, put her finger on the spot, when after inquiring into the affairs of the Nurses' Co-operation whilst in England, she reported to the International Congress at Buffalo: "Those members of the society who have been constantly present at all the meetings, finding the power in their hands, have grown accustomed to using it, and have (possibly unconsciously) come to believe that they are the only persons qualified to manage the business of the Association." This was exemplified also by the fact that Dr. Voelcher became a member of the Co-operation and a member of the Committee of Management all within the course of the last year; in the same way as in the previous year Dr. Wallace became at once a member and a member of Committee. There is also an "honorarium" of £60 to the "medical officer,' which is a new feature, and from which we conclude that the hon. physician and hon. surgeon are only consultants. The nurses do not receive "free medical treatment" under these circumstances.

For the rest the report shows 497 nurses on the general staff, and 20 special nurses for mental cases. The receipts are excellent and show a good balance at the bank. The Club seems to be working much better, and the debt on it has been paid off—out of the General Fund apparently. The club and the new flat at Vandyck Mansions were, however, both run at a loss. The accounts show a loss of £330 on the club, and a loss of over £100 on the flat. The appointment of Miss Laura Baker to be home sister at the club is very popular, and perhaps by next year there may be a different tale to tell. With so many good points and excellent aims the co-operation ought to be one of the leading nursing organisations in the world; it ought to be foremost in expressing an opinion on all nursing questions, and a leader of public feeling in these matters. It ought to send a representative to the Fourth International Congress of Nurses at Berlin this summer; it ought to be ambitious of more than money, it ought to accept the onerous duties its success entails, and help forward the organisation of the nursing profession in every way. The members of the Co-operation, or the more advanced amongst them, should be encouraged to acquire a knowledge of business and to speak, so that they may be able to secure for their sisters, who they could then represent with power and authority, many things which they lose under the present unsatisfactory and easy management.

### Lectures on Ophthalmic Nursing.

By A. S. Cobbledick, M.D., B.S.Lond., Senior Clinical Assistant and late House-Surgeon and Registrar to the Royal Eye Hospital.

### LECTURE XXVIII.—OUT-PATIENT HOSPITAL WORK—SIGHT TESTING (*continued from page* 258)—FOREIGN BODIES AND ACCIDENTS.

This is an important branch of ophthalmic nursing where much work has often to be done in a limited time, and patience and care are required at every turn.

A great variety of cases are met with, so that nurse and student must be fully alive to some of the risks which are being constantly run. The following are some of the most important points to be remembered:—

1. Cover all cuts or abrasions on the fingers or hands with a collodion dressing.
2. Carefully disinfect and wash the hands after examining any septic or specific case.
3. All instruments that have been used should be cleansed in cold water and sterilised by boiling.
4. Exercise great care in separating the lids in a case of ophthalmia neonatorum: the infective secretion accumulates and under such a pressure that when the lids are separated the discharge is expelled to a considerable distance and may find its way into the attendant's eyes. In hospital practice where a nurse has often to attend to several each morning, she should wear large circular spectacles; if not, she should lessen the risk run by separating the lids at arm's length and not with the face immediately over that of the infant.
5. Never use drops from a bottle which is not clearly labelled; never give the surgeon a drop bottle without first seeing distinctly what its contents are; the surgeon likewise should never use drops handed to him without first noting the contents for himself. In this way the risk of making a dreadful mistake is minimised.
6. If the tubing of the drop-bottle is suspected of infection, it should be removed and sterilised.

*Foreign Bodies and Accidents.*—The former are quite common and vary in severity of symptoms, to which they give rise, from a mere irritation to very severe pain. The most common are small pieces of grit, coal dust, fine pieces of steel, iron or emery, lime and hot metal. If the foreign body is lying loose in the conjunctival sac it can be readily seen and removed.

The examination is never complete until the upper lid is everted; it is on the ocular surface of the upper lid, a short distance behind the margin, that most particles in the conjunctival sac seem to eventually lodge. With every movement of the lid the particle comes in contact with the sensitive cornea, causing very considerable pain and lachrymation.

If a foreign body remains in this position for some time, it produces a peculiar pinkish coloration of the sclerotic around the cornea which simulates the appearance found in slight attacks of cyclitis.

At times the foreign body is embedded in the ocular conjunctiva, and as the conjunctiva is not very sensitive, the amount of discomfort is not great.

Foreign bodies impacted in the cornea are commonly met with in hospital practice, especially pieces of iron, emery, and steel filings. They set up great irritation, and if not removed early the circumcorneal vessels become much injected, and some degree of iritis supervenes. If such a foreign body is not removed, the corneal substance around is softened so that it becomes loosened and in time is shed by the movement of the lids over it.

The worst class of foreign bodies on the conjunctiva are those which have a caustic action in the form of lime, usually mortar or plastering. Steam and hot metals produce bad burns. The extent to which vision is subsequently affected largely depends on the damage done to the cornea. If the conjunctiva is extensively burned, much trouble results from the union of the palpebral and ocular surfaces, and the formation of cicatricial bands.

*Treatment.*—There is little difficulty in removing foreign bodies other than those on the cornea. Those on the ocular or palpebral conjunctiva can be readily removed with a piece of damp wool or lint.

It is most important to remember that if any *instrument* is used to remove a foreign body, all manipulations should be made from *behind* the patient with his head firmly fixed.

Foreign bodies impacted in the cornea require great care in their removal; the cornea should be first thoroughly anæsthetised with cocaine; the next point of importance is to have a strong light thrown on the area of operation. The blunt-pointed spud so much in use is a very dangerous instrument, for if the foreign body is small, considerable damage may be done to the corneal substance in removing it.

It is better to use an arrowheaded needle and so pick up the foreign body without injuring the corneal epithelium around.

Burns with lime require special treatment; in most cases if the patient is in great pain and cocaine does not have the desired effect, a general anæsthetic should be given.

This allows of a most searching examination and the removal of every particle of lime; the sac should then be well irrigated with a weak solution of acetic acid and castor oil drops containing cocaine should be instilled every two hours; iced boracic pads give relief and diminish swelling of the lids.

If the cornea is much affected the prognosis is serious as complete opacity is likely to supervene.

*Perforating Wounds of the Eyeball.*—These unfortunately are rather common, and the results may be of such a serious nature that great care and judgment are always necessary in treating them. The perforation is not always apparent, therefore any *history* of an accident should entail a very careful search for a wound; one of the best and surest signs of a perforation is the lowering of tension. If the wound is situated behind the ciliary zone a small bead of vitreous often presents.

When the perforation can be seen, the points to determine are?—Is the body which has caused the perforation in the eyeball? Is the lens injured?

If the body is in the eyeball is it impacted in the sclerotic or is it lying in the vitreous?

The first question is not always as easy to answer as might be imagined: sometimes it can only be definitely settled by an *x*-ray photograph.

If the lens is injured the aqueous is given access to the lens substance and opacity results in a few hours' time.

Wounds at the edge of the cornea and involving the ciliary region are especially dangerous on account of the possibility of a sympathetic inflammation in the sound eye.

Many badly-damaged eyes may be saved with much-diminished vision; frequently the lens has to be evacuated; in some cases an iridectomy is necessary, and in most cases the magnet must be used to remove the projectile, which is usually metal.

If some months after a wounded eye has been saved, the patient comes up complaining of irritation and photophobia in the sound eye, the onset of sympathetic inflammation should be suspected. This is a serious affection which may end in loss of the sound eye, therefore the question arises at the time of injury, Is it better to lose the injured eye and so run no risk of sympathetic trouble in the sound eye at some later date ?

This is but a brief outline of an important subject which always requires experience and sound judgment to deal with it successfully.

### The Nurses of Leavesden Asylum for Imbeciles.

INTERVIEW WITH THE MATRON. BY OUR COMMISSIONER.

If there still lingers in the public mind an impression in some quarters that hospitals for the mentally afflicted are little better than dreary prisons, anyone who has paid a visit to the institution at Leavesden, a charming village in Hertfordshire, easily accessible from Watford, is in a position to remove it. Situated on an eminence, and surrounded by large and admirably laid-out grounds, the interior of this great asylum for imbeciles, under the jurisdiction of the Metropolitan Asylums Board, with accommodation for not far short of two thousand patients, much more nearly resembles a well-appointed and up-to-date general hospital than a prison. This applies especially to the wards on the infirmary floors, but the wards in the general blocks are also extremely light, airy, and cheerful. Patients suffering from tuberculosis have the advantage of pleasant outside shelters; outdoor exercise is freely taken by the inmates; there is a splendid recreation room with an excellent stage; and every possible effort appears to be made to ameliorate the lot of the unfortunate individuals whose state of mind renders their detention necessary. Since the present medical superintendent, Dr. Elkins, and the matron, Miss E. M. Howell, were appointed between five and six years ago, there have been various improvements in the administration, and both these officials are entirely in sympathy with the movement for the elevation of mental nursing. The matron is able to speak with the authority of varied experience. Trained at

THE NURSES' RECREATION ROOM AT LEAVESDEN.

Bristol General Hospital, she had spent nine years in general nursing and three years in mental nursing before she came to Leavesden After she had been kind enough to accompany me through several of the wards, and show me the pretty chapel, the commodious kitchen and store-rooms, as well as the recreation-room belonging to the nurses, I asked her to tell me some particulars about the staff and the system

"Of course," she said, "I am not responsible for the male side of the asylum, on which all the officials but one are men. The exception is a superintendent nurse, who is in charge of the male infirmaries."

### THE NURSES OF LEAVESDEN ASYLUM FOR IMBECILES—*Continued.*

"We will therefore leave the male side out of the question for the present. But I conclude that there is also a superintendent nurse in charge of the female infirmaries?"

### The Infirmaries and General Blocks.

"Yes, and both superintendents are hospital-trained nurses. There is an assistant matron, who has been here over twenty years, and a night superintendent. Also there are on the female side two head attendants who hold the certificate of the Medico-Psychological Association. The number of day nurses is 60, and of night nurses 18. This would have to be larger, but a certain amount of work is done by the patients"

"Does that apply to all departments?"

"Practically. For instance, there is a laundry department with a superintendent, 13 maids, and four men; and with the laundrymaids there are 40 patients working. The housemaids are helped in the same manner, and as you saw just now, several patients are employed in the needle-room. In the wards themselves a great deal of needle-work is done, and the nurses get as many patients as they can to assist them in their duties."

"How is the nursing in the infirmaries arranged?"

"For each of the four floors containing 50 patients there is a charge nurse, a deputy charge nurse, third and fourth nurses, and sometimes a fifth. The other two floors are devoted to tubercular cases, and contain 39 patients each."

"And the general blocks?"

"There are three general blocks with 150 beds each, one with 110 beds, and one with tubercular patients well enough to be about. With the exception of the ordinary general block containing 110 patients, in which five nurses are on duty, the whole of these have six nurses each attached to them. One of the changes initiated since I came here was the increase of night supervision. The night superintendent visits the wards in succession, and helps to quiet the noisy patients, and soothe those who cannot get rest."

### Hours of Duty.

"When do the day nurses go on duty?"

"At six in the morning. Breakfast is from 8 to 8.30, and 8.30 to 9. The nurses have a light lunch in the wards at their convenience, and dinner is from 2 to 2.30 and 2.30 to 3."

"Is not that rather late?"

"Yes; but the nurses cannot leave the wards for their dinner until all knives used by the patients have been taken away and locked up. Tea is going from 5 to 6.30, and nurses have it in detachments. Supper is after 8 when they are off duty. From 8 to 10 the nurses can go out as they please."

"Are they not off duty at all in the day?"

"They have one day off every week, and occasionally, if they prefer, they are allowed to put two days together and go out for the night. The night nurses go on duty at 8 P.M., and have a meal in the night when they can, consisting of an egg and bread and butter, or jam, and a cup of tea. They breakfast at 6.15, and have to be in their bedrooms at 7.30. At 3.30 P.M. they are called, have tea at 4, go out, if they please, until 7, when they have a good dinner. We have tried several hours for the night nurses' dinner, and find that they enjoy it most at 7. They are off duty one night a fortnight, when they are allowed to stay out. We make arrangements to drive nurses to Watford Station, and to bring them back, when they desire to go by train. The distance is four miles."

"How about holidays?"

"The first-year nurses have twelve days annually, and the second-year nurses fourteen days. As to remuneration the salary on joining is £18 a year, rising to £23, with board, lodging, washing, and uniform. If promoted to be charge nurse, the salary is £25, rising to £31 a year, with the same emoluments. An annual payment of £2, payable quarterly, is made, in addition, for good conduct. If desired, a money payment of £2 10s. per annum will be allowed in lieu of beer or equivalent beverages. Nurses employed on night duty receive an additional £4 per annum. Uniform for indoor wear is provided and also outdoor uniform when the nurses have to be out of doors on duty with patients."

### A Nurses' Home.

"The accommodation for them at present, I gather, is quite inadequate?"

"Altogether so. That is why a nurses' home is being built. As three nurses have frequently to sleep in one room, it cannot be said that public money is being wasted on the Home. The latter will contain 40 bedrooms, a good recreation-room, and bath-rooms."

"You have a handsome recreation room now?"

"Yes, and we shall probably convert that into a work-room. Meals will continue to be taken in the main building as they are at general hospitals."

"When do you expect the Home to be opened?"

"Some time during the summer. It will, I believe, be connected with the main building by a covered way."

### The Question of Recreation.

"Do you do much in the shape of providing recreation for the nurses?"

"Books are supplied from the library by the chaplain, and table tennis, bagatelle, cards, and draughts are provided in the sitting-room. There are occasional subscription dances, arranged among the staff, which are greatly appreciated. Speaking of dances, once a week through the winter the patients have a dance from 7.30 to 9.30 in the recreation hall, and at Christmas there is one on a larger scale, with an interval for tea and coffee. Concerts are also occasionally got up by the officers and nursing staff for the patients, Mrs. Athelstan Clark acting as accompanist. There is a bicycle shed, and in the summer the nurses do a good deal of cycling. There is also a tennis court."

### The Training.

"Is it indispensable that the nurses should receive two years' training?"

"Two years and three months if they wish to obtain the certificate of the Medico-Psychological Association. This is rapidly becoming essential, and no nurse who does not pass her examination is promoted to the position of charge nurse. The course of training begins with demonstrations on nursing, pure and simple, by the superintendent nurses. These are followed by lectures on anatomy, physiology, and diseases by the first and second medical officers; and by lectures on insanity by the medical superintendent. The third medical officer also instructs those who wish, to obtain the First Aid Certificates of the St. John Ambulance Association."

"You are strongly in favour of two years' training?"

"Very strongly, and also I think that all mental nurses should have one year's hospital training. This is not practicable yet, but it is, as I stated in our magazine, my ideal."

### The Hospital Nurse.

"And supposing that the hospital-trained nurse wants to take up mental work?"

"She should be allowed to obtain the Medico-Psychological certificate at the end of one year instead of at the end of two. But one year's training is most requisite. Too much stress cannot be laid upon the fact that in an institution for the insane the main nursing is mental. I say this, because there is a tendency to underrate the importance of mental training."

"Even in the infirmaries," continued the Matron, "the hospital nurse will soon find there are differences. A poultice or dressing, which should be changed every four hours, may have to be renewed every hour, because the patient pulls it off. Medicines should be given, and temperatures taken, at regular intervals, but this is sometimes most difficult to carry out, the patient being excited and troublesome. The tactful nurse will leave the patient for a time, then try again and again, until she is successful. It is much more difficult to keep our patients free from bed-sores than is the case with patients in hospitals. Our nurses manage wonderfully well in this respect. Everywhere, again, knives, scissors, medicines, powders, and lotions have to be locked up. The doors of the wards have to be kept locked; and hot water keys to be looked after lest the patients should scald themselves. Most necessary of all, there must be strict supervision of the movements of the patient, without it being apparent that she is watched."

"I conclude that you are now alluding to precautions in ordinary cases rather than acute cases?"

"We seldom have acute or suicidal cases. Our staff is not large enough to cope with them. They require such close supervision that nurses in attendance are rarely on duty with them more than two hours. When one of our patients begins to show suicidal tendencies she is transferred to an asylum for acute cases.

### THE MOST IMPORTANT POINTS.

"What do you consider are the most important points of mental nursing in the treatment of ordinary imbecile cases?"

"One primary object the nurse should always set before herself is to rouse the patients to occupy themselves. Employment is a great factor in treatment. A patient can often be coaxed to employ herself. The promise of a new frock will tempt her to make one. Our patients are more stupid than violent, but they have many bad habits. It is a part of mental nursing to cure these habits. Troublesome patients can also be soothed by gentle persistence, and noisy ones can be induced to be quiet. If the normal intelligence cannot be restored, the normal standard of behaviour can be improved."

"Are the nurses frequently with the patients out of doors?"

"They go out for walks with them, and attend them if they are transferred to another asylum. The medical superintendent can also give a nurse leave to take a single patient out, in which case the nurse wears her private dress. This is a privilege which the patients value highly. Nurses attend the patients at the services in the chapel, which take place twice daily and thrice on Sunday. Some of the nurses and attendants form the choir, and the chaplain is choirmaster."

### VITAL CHARACTERISTICS OF A MENTAL NURSE.

"Finally, Miss Howell, what do you regard as vital characteristics for a capable mental nurse?"

"She must be a good disciplinarian, but she should never lose her temper. She must be very orderly, but she should not be disturbed if as soon as she has put things in order they are all disarranged again. She must be very kind, and yet make the patients do as they are told. She must be willing to be responsible for the clothing and bedding, but she should be prepared to smile when a patient throws things out of the window or destroys her clothes. She must be willing to listen to the same story or complaint times without number; indeed, her patience should be simply endless. If a hospital nurse should be a good woman, an asylum nurse should be an angel. Of course, I have never met such paragons, and I hope I never shall; for if I find one, it will be time for me to resign my post to her. But when people talk as if mental nursing were inferior to hospital nursing, I venture with equal experience of both to affirm that it is more difficult, and ought to stand first. The diseases of the mind are, surely, more terrible to deal with than those of the body!"

### The Preparation and Application of an Abdominal Fomentation.

### EXAMINATION QUESTIONS FOR NURSES.

THE question was as follows:—"Describe the arranging, preparation and application of an abdominal fomentation."

### FIRST PRIZE.

In arranging for an abdominal fomentation I should begin by putting a kettle of water on to boil, and then get the necessary articles placed on a table by the patient, *i.e.* a basin, fomentation flannel—two or three folds thick—a wringer or towel, a piece of jaconet or mackintosh—whichever is obtainable—which should overlap the flannel all round to prevent the patient's clothes and bedclothes getting wet, also to keep the flannel moist and the heat in. A piece of absorbent wool large enough to cover the jaconet, turpentine, or liniment if any has been ordered to be sprinkled on the flannel, a many-tailed bandage and safety pins. For preparation I should place the fomentation flannel in the wringer, or towel, and put them into the basin with the ends of the wringer or towel overlapping the edge of the basin. Now proceed to get the patient ready by uncovering the abdomen and placing the bandage under the back, covering the patient again loosely with the bedclothes, in order that no time may be lost when the fomentation is ready, also to avoid exposure or chill. Next, when the water is quite boiling, pour it over the fomentation flannel and wring out very quickly and tightly. Quickly that it may not get cool; tightly to leave the flannel as dry as possible and so avoid blistering the patient's skin. If turpentine or liniment is to be used, sprinkle the quantity which is in readiness over the flannel. Now turn down the bedclothes and apply the fomentation gradually to prevent too much shock or burning the patient, but at the same time avoid delay and encourage the patient to bear it as hot as possible. Cover over with the jaconet, or mackintosh, then the absorbent wool, and fix with the many-tailed bandage and safety pins, and replace the bedclothes. The fomentation should be changed every hour, until relief is obtained.

NURSE BETTY.

### SECOND PRIZE.

To apply an abdominal fomentation I should get together all the necessary things, such as follows:—A kettle of boiling water (to be kept on the boil), a bowl, a wringer, safety pins, jaconet, or oil-silk, wool, a binder or many-tail bandage. The three latter I should put to the fire. If there was an abdominal wound I should apply boracic lint fomentation, three thicknesses; otherwise, flannel, two thicknesses. After making all the necessary arrangements, I should next prepare my patient by placing her on her back, if possible, roll up the night-dress all round to the waist, and gently wash the abdomen with hot water and soap. If a wound, I should use some antiseptic lotion instead and apply a sterilised swab until the fomentation was ready to replace it. I should then cover my patient with a blanket, and slip the binder or many-tail smoothly under the back, and next prepare the fomentation by rolling it up in the wringer and pouring boiling water over it. Allow the fomentation to soak a few seconds, then wring it out absolutely free from the last drop of water. If a very large fomentation, it is better to have sticks through the wringer and get assistance in wringing it. Then apply to the abdomen as hot as can be borne, cover quickly with the warm jackonet and wool, and secure with the binder. Care must be taken in cases of paralysis that the patient is not blistered by over heat.

BELINDA.

### THE SUCCESSFUL PAPERS.

It is cheering to be able to say that the papers this month are a great improvement on those sent in for December. There are several excellent answers, so that five honourable mentions have been awarded as well as the two prizes. The first prize is gained by "Nurse Betty," whose arrangements are all good, with the exception, perhaps, of that relating to the preparation of the fomentation within sight of the patient. I think it would be preferable to do it, if possible, in another room, as patients, often timid and nervous, would not then see how hot it was. "Nurse Betty" mentions the important and most sensible precaution of applying the hot flannel gradually. It is wonderful how hot it can be borne if let down on to the skin slowly, and the nurse's hands quietly and leisurely withdrawn by degrees. It is only a rough and inexperienced nurse who contents herself with the inadequate test on her own elbow and then dumps the steaming instrument of torture on to the sensitive skin with a curt "It is not too hot and must be borne." "Belinda," the winner of the second prize, very wisely gives warning of the ease with which paralysed patients may be blistered.

### HONOURABLE MENTION.

This is gained by "Devon," "Brum," "Tally," "Mrs. Podger," and "Cleriss." "Devon," who sends an excellent paper, would have received the first prize, but she divided her answer into innumerable headings, thereby unfitting it for publication in our limited space.

### QUESTION FOR FEBRUARY.

Define the different degrees of danger to life from various kinds of severe burns and scalds and the means you would employ to obviate fatal results.

N.B.—You are not asked how you would treat the burns themselves.

THE EXAMINER.

### RULES.

The competition is open to all. Answers must not exceed 500 words, and must be written on one side of the paper only. The pseudonym, as well as the proper name and address, must be written on the *same* paper, and *not on a separate sheet.* Papers may be sent in for fifteen days only from the day of the publication of the question. All illustrations strictly prohibited. Failure to comply with these rules will disqualify the candidate for competition. Prizes will be awarded for the two best answers. Papers to be sent to "The Editor," with "Examination" written on the left-hand corner of the envelope.

N.B.—The decision of the examiner is final, and no correspondence on the subject can be entertained.

In addition to two prizes honourable mention cards will be awarded to those who have sent in exceptionally good papers.

### Locked in a Mortuary.

### THE EXPERIENCE OF A NURSE.

I WAS night nurse in a scarlet fever ward in an infectious hospital, and, as we had several bad cases in, it was no surprise to me when going on duty one night to be told that one of our boys had died during the afternoon. The probationer was off duty, so the sister was alone, and stood giving me directions, holding a small piece of paper in her hand, on which was written the name and age of the dead child. She had forgotten to pin it on the corpse, as was the custom, for purposes of identification, so she intended calling there before going up to supper. She stood chatting, and before either of us thought, the bell rang, and as she was head sister, and carved at table for the day nurses, she dared not be late for supper; so, hastily pushing the paper into my hand, she said: "You could easily slip up with it when matron has been her round," whilst I, knowing it was a breach of discipline, did not mind obliging her, as she was my senior, both in years and training. "There are two others in," she said, "but as they are adults you cannot make a mistake," and, saying good night, she went off duty.

### THE PATIENTS.

The matron usually came her round about 10 P.M., called at all the twelve wards, and was back up again by 10.45 P.M. My ward was situated halfway between the six top wards, and the five lower ones; and close to me were the laundry, the south lodge gate, and, in a little enclosure, surrounded with hedges and rhododendron bushes, the mortuary. I waited till I heard the matron's crunch, crunch, on the gravel in front of my ward, on her way back to the house after completing her round, then took a glance round at all my patients. I had 34 beds, but 4 were vacant, so there were 30 lads of all ages from 7 to 20 years. Some were fairly on the way to recovery, others in the first stage, but all what we called "acutes," *i.e.*, too ill to be called convalescent. They all seemed comfortable, so I replenished the fires, then knowing our resident doctor was at a dinner-party, and would not do his round much before 1 A.M., I felt pretty safe to leave my ward, quite confident that in less than ten minutes I should be back again. So taking a taper and a match-box, I started. I ran to the mortuary, and as the key was always in the outside of the door, I lit my taper, and had no difficulty in entering.

### IN THE DARK.

I remember noticing that there was only one match in the box, but as it lit my taper I did not mind. I pushed the door and went in, and almost immediately a draught from the skylight blew my taper out. At the same time the door, which was on a swing hinge, closed behind me, and I realised with a dread chill at my heart that I had forgotten to remove the key from the outside, and as there was no handle, nothing but a keyhole in the door on the inside, I was locked in! I felt my way along the slabs, six there were, till my hand touched the feet of the man from one of the top wards who had died of diphtheria; then, further down, was another corpse—a girl about 17—from the typhoid ward, and lastly I came to a tiny one, which I knew must be my little patient. Poor little Tom I thought as I pinned the paper on. He had no father and his mother drank, and he only sold matches in the street and was very often cold and hungry, he had told me, and was so ill when he came to us that we never had much hope that he would recover. Then I set to work thinking how I might get out. Perhaps, if I shouted loudly enough, one of the nurses in the lower wards might hear me, but it was very unlikely that they would guess where the voice came from, and as to searching the mortuary, well, judging from what I should have done myself, no one would ever think of going there at 11 o'clock at night.

WAITING FOR THE DOCTOR.

We were all inclined to be a little superstitious, and would not go there from choice. I stood on the end of the slab, on which the man rested, and tried to spring at the skylight, which was almost a foot out of my reach. I thought I might possibly cling to it, and draw myself up, and once on the roof, I would find a way somehow to get down, though it was fairly high. But alas! for my hopes; my first spring ended in my falling backwards across the feet of the corpse, and from there to the floor, catching my head on the corner of the slab. This was a caution not to try again; I felt that I might easily split my head open if I fell awkwardly. So I seated myself on a vacant slab, and folded my arms. There was nothing to do but wait till the doctor came his rounds, as he would pass close to me on his way to the lower wards.

A CALL FROM THE WARD.

I had sat about an hour as it seemed to me. I had my watch on, but I could not possibly see the time, when all at once I heard someone shout "Nurse!" I listened with every nerve strained. Again and again the call came. It was from my ward! I sprang to my feet, and made my way to the door. Inserting one finger in the key-hole, I pulled and tugged with all my strength, but it was immovable—I was helpless! I stood almost paralysed. Again one of my boys shouted, "Nurse, I want a drink," and others chimed in, till to my already shaken nerves it appeared as if everyone in the ward was awake wanting me, and I was unable to go to them. Would they get out of bed and fall in the fire, or wander out into the cold? I tortured myself with these thoughts, then sat on the floor, buried my head in my apron, and began to cry.

A WEIRD SCENE.

How long I sat I do not know, but the stone floor had chilled me through, for the month was March, and it was very cold. I got up stiffly, and sat on the edge of the slab again. The voices had ceased calling, only I fancied I heard some-one moaning, and cries seemed to come from somewhere, but perhaps it was only my fancy. Would doctor never come? The moon had evidently risen, for its faint rays came through the skylight, and I could just see the corpses dimly outlined in the moonlight. It was very weird, and must have been long past midnight, but I was not frightened. There was nothing to fear but the infection from those dead bodies, and nurses in infectious hospitals think nothing of that. I began to say the Lord's Prayer aloud for company, and had got half-way through when I found I had forgotten it! The idea of forgetting a thing I had been in the habit of repeating from childhood made me laugh, and the laugh in that dreary place seemed quite awful.

A SCREAM FOR HELP.

I began to pinch my arms, to try and get some warmth into them, but they were too numb with cold, and all this time I was in a veritable agony about my patients. Suddenly I sprang up! I fancied I heard the doctor's step, coming from my ward. Was it he? Yes, surely, he was walking quickly on the gravel. Angry no doubt with me for being absent from the ward, and expecting to find me gossiping with the nurses in the lower ones. He was nearly opposite the mortuary, when I scrambled to my feet on the slab, and putting my two hands to my mouth to make the sound go further, yelled at the top of my voice at the skylight, "Doctor, I'm in the mortuary." He paused, and I thought I heard him say, "the Devil!" Then he went on again, thinking no doubt that he must be mistaken. Almost frantic with fear that he would not release me, I screamed with all my strength, "Doctor, doctor, come to the mortuary," and at last had the satisfaction of knowing that he had heard me, for I heard the click of the gate which led into the enclosure, and then the key turned in the door.

RELIEF AT LAST.

The joy and relief at being free to go to my ward were so great that at first I could only whisper to myself "Thank God, thank God!" The doctor entered, and striking a match lit my taper. "What does this mean?" said he, sternly. Gradually I made him understand how I came to be there. He returned with me to the ward, and making me sit down, lit all the fires which had gone out, and attended to the patients, although I assured him that beyond being chilled through and through, I was all right. He said that when he came in first and found that the midnight temperatures had not been charted, he knew I must have been absent from the ward for some time, and nothing but the predicament he found me in could have saved me from being dismissed. The patients were none the worse as it happened, for being evidently tired out with calling they had fallen asleep again in more or less uncom-fortable positions.

THE SISTER'S CRITICISM.

When the sister came on duty at 8 A.M., and I told her my adventure, all she said was, "Well, you are a fool! Fancy taking a box with one match in it, and forgetting to remove the key from outside, any probationer in her first year would know better than that!" She got severely censured by both doctor and matron for breaking the rules in telling me, a junior nurse, to leave my ward in order to complete work which she had left unfinished, she was consequently always disagreeable with me from that day till I left the hospital, twelve months later.

### Appointments.

MAIDENHEAD UNION INFIRMARY.—Miss Florence Grey has been appointed assistant nurse. She was trained by the St. Peter's Sisters at Mortimer Road, Kilburn.

MCKELVIE ISOLATION HOSPITAL, OBAN, N.B.—Miss Mary Brown has been appointed matron. She was trained at Glasgow Western Infirmary, and has since been charge nurse, and acting night superintendent, at the Fountain Fever Hospital, Lower Tooting, London, and house matron at the Banstead Road Schools for Ringworm, Sutton, Surrey.

NORFOLK AND NORWICH HOSPITAL.—Miss F. A. Cann has been appointed lady superintendent. She was trained at the Westminster Hospital, and has since held appointments as ward sister at the Hospital for Women, Soho Square, London; night superintendent at University College Hos-pital, London; temporary matron at Highgate Infirmary, and assistant matron at the Grove Hospital, Tooting, S.W.

WALLSEND AND WILLINGTON QUAY JOINT FEVER HOS-PITAL.—Miss Margaret Schweppe has been appointed matron. She was trained at the Royal Infirmary, Newcastle-on-Tyne, and has since held the positions of ward sister and night superintendent at the same institution. She has also been matron of the Private Hospital, West Hartlepool, and sister-in-charge of the Nurses' Home, Haworth, Keighley.

### Death in Our Ranks.

WE regret to hear of the death, on February 9th, of Nurse Margaret Irvine, of the Royal Scottish Nursing Institution, Edinburgh. She went early in January to Lochborsdale, South Uist, to nurse during an epidemic of typhoid, and more particularly to attend on a fellow-nurse who was suffering from that disease, but recovered. Miss Irvine was for four years at the Belvedere Fever Hospital, Glasgow.

### Everybody's Opinion.

### ENEMA RASHES.

"N. AND L." write: Practical observation will show even the youngest probationer that erythema, or the enema rash, is a very common occurrence, with both adults and children, after a simple soap-and-water enema. It is entirely out of the question to suppose that this is a newly-arisen complication, and any nurse possessing ordinary powers of perception will know that this is the case. We have had many years of experience in our profession, and recall this circumstance from the first years of our probation.

### PRIVATE NURSING IN DAVOS PLATZ.

MISS GEORGINA PRYKE writes from the British Nurses' Home, Davos Platz, Suisse:—I have seen several answers in THE HOSPITAL to nurses, asking for information about private nursing in Davos Platz. You give the Davos Invalids' Home for them to apply to. The Invalids' Home is for patients only, and there is no staff of nurses kept there. Private nursing is done from the British Nurses' Home, Davos Platz. The Institution of Nursing Sisters, 4 Devonshire Square, London, sends out nurses as required to the home in Davos, as it is found to work better, the season being very short and the number of nurses needed very uncertain. Unless a nurse has means, it is no use trying to start private nursing, on her own account, as living is very expensive here. I send this information as I thought it might be a help and also to spare the matron of the Invalids' Home, the trouble of answering or sending the letters to me to do so.

### SIR WILLIAM BENNETT ON PRIVATE NURSING.

"A MATRON" writes: I should be sorry indeed to think that Sir William Bennett's words were meant to imply what "A Frequent Contributor," in your issue of February 6th, suggests, viz: that a nurse should do anything to outrage her modesty, or that of her patients, or lower her womanhood. I would rather think that they were intended to mean what "A Frequent Contributor" herself admits:—That there is a necessity that knows no laws, and that nurses should be able to rise to such occasions, and do what is required in a professional manner. "A Frequent Contributor" says she has been nursing ten years, the last seven in the male wards of civil and military hospitals. If that is so, and she has had a full three years' training in hospital, she cannot have had any experience as a private nurse, and it was, I believe, principally to those about to become private nurses that Sir William Bennett's remarks were addressed. Therefore, if she will forgive me for saying so, I do not think she is in a very good position to judge of the matter; as hospital nursing and private nursing often differ widely. Therefore I am of opinion that every nurse should do a certain amount of private nursing and consider it part of her training. A doctor in private practice has often to do many things for female patients which if they were in hospital he would for his own sake and theirs gladly leave in the hands of trained sisters and nurses. Even so, a private nurse may find it necessary to do many things which she would not be required to do in large hospitals, because there are plenty of men there to do them. I think in such cases that a nurse who quietly rises to meet her patient's need in a true christian and professional spirit does not in any way outrage her own modesty, or that of her patient, or lower her womanhood. Before I became a matron with nurses under me I had a good deal of experience with male patients. In the wards of a large training school, in private nursing, and in a male hospital with only visiting doctors, and have nursed almost all kinds of cases. And I can safely say that only on the most rare occasions have I ever been called upon to do anything that I could not do with as much delicacy, and almost as little exposure, as giving a "blanket bath." I think a great deal depends, not so much on "what" the nurse does, as "how" she does it. At the same time, doctors ought not to send nurses to such cases, when they know the patient to be of low moral character, and suffering from the consequences, unless it be absolutely necessary. For such cases they should endeavour to secure the services of a male attendant; and if there are not enough male nurses at present to meet the demand, then the sooner more are trained the better. In the meantime doctors should relieve the nurses of as much of the unpleasant part of the work as possible. And the nurse must try to lose sight of the degraded "man," and see only the "patient," or I must admit it would be difficult to keep a natural feeling of disgust and loathing for such a creature entirely in the back-ground. The nurse would also do well to remember that the safe disposal of soiled dressings, disinfecting of linen, etc., is safer in her hands than it would be in the hands of some member of the household, who perhaps would be quite ignorant of the nature of the disease from which the patient is suffering, and also of the danger of contamination to others. Looking at it from that point of view I think many nurses will agree with me that even under such very unpleasant circumstances a nurse might do good work in safeguarding the innocent, and so prove her womanhood to be of a high and noble order instead of in any way lowering it. But I do not think that the question is one which the average nurse needs to trouble herself so much about, because if she is attached to a properly organised institution she will have a fully-trained matron to refer to in any case of doubtful propriety, who should be able to judge each case on its merits, and relieve the nurse of all responsibility. If, on the other hand, she is nursing on her own responsibility she can always decline such cases. Therefore I fail to see where much difficulty need arise, except in the lower order of institutions where the matron is untrained, and therefore unable to understand and sympathise with her nurses in their work. Yet I feel it only right to add that I think a great deal of unnecessary discussion—and also unseemly discussion—is often brought about by a certain type of nurse who is always ready, in season and out of season, to discuss what might be termed the unpleasant details of her work. I think it often arises from a desire to pose as a martyr to circumstances, or to show herself off before her fellow-nurses and others. This she often does to an extent she little dreams of, and with anything but credit to herself, her training school, or her profession generally. One cannot help feeling that such women have missed their vocation in becoming nurses, and the sooner they settle down to their work and do it in a right spirit, or take up other work which they may be better fitted for, the better it will be for the profession. It is generally nurses of that type who like to regale their friends, and anyone else who cares to listen to them, with what seems to them ghastly accounts of operations, accounts which get passed on to others, often with additions, and which fill the hearers' hearts with—perhaps unnecessary—fear, if at any future time they or their friends may have to undergo an operation. If such nurses were eliminated from the profession I feel sure we should hear a good deal less about indecent cases, for I have generally found such nurses guilty of gross exaggeration and cruel misrepresentation.

### Presentations.

GROVE HOSPITAL, TOOTING.—Miss F. A. Cann, on resigning her appointment as assistant matron at the Grove Hospital, Tooting, was presented with a silver tea service and silver tea-knives from the matron, the medical and nursing staff, and a silver-plated hot-water jug from the domestic staff, as well as some individual presents from the nurses and servants.

EASTERN HOSPITAL, HOMERTON.—Mrs. S. E. Carr, charge nurse for ten years at the Eastern Hospital, Homerton, has been pensioned off on account of ill-health and has been presented with a purse of gold and a portmanteau by her fellow charge nurses, who on the occasion of the presentation said that they could not let her go without asking her to accept the gift as a token of their esteem.

SAMARITAN FREE HOSPITAL. — Sister Williams, after being on the nursing staff of the Samaritan Free Hospital for thirty years, is retiring on a pension. The medical staff presented her with a purse of money; while the matron, nursing staff, and domestic staff gave her a serviceable gift of house linen. In addition she received numerous useful presents from the secretary and ladies connected with the hospital.

### Echoes from the Outside World.

### The Movements of Royalty.

IT is announced that their Majesties the King and Queen will hold a Diplomatic and Official Court at Buckingham Palace on Friday, March 18th, at 10 o'clock P.M., and another Court on Friday, March 25th. In connection with the marriage of the Princess Alice of Albany with Prince Alexander of Teck, the King has made a number of promotions in, and appointments to, the Royal Victorian Order, including the bridegroom, who becomes Knight Grand Cross. Viscount Gough, Minister at the Court of Saxony and Saxe-Coburg Gotha, and Sir Robert Collins, Comptroller of the Household to the Duchess of Albany, are created Knight Commanders. The King and Queen will visit Cambridge on March 1st. An official programme has been issued by the authorities of the University.

### The Royal Guests.

QUEEN EMMA of the Netherlands paid a visit to the Netherlands Minister, in London, on Saturday last. She was previously received, on her arrival at Waterloo Station, by a large contingent of members of the Dutch colony, who cheered her enthusiastically. Queen Emma accepted a bouquet of orchids presented to her by the daughter of the President of the Dutch Society in London. She then made a number of purchases at West End shops. After luncheon at the Netherlands Legation, at which the Foreign Secretary was present, an address, signed by a large number of London Netherlanders, expressing loyalty and devotion towards the mother of Queen Wilhelmina, was handed to her. On Monday Queen Emma visited the Wallace Collection and the National Gallery. Other Royal guesss, including the Queen of Würtemberg, visited the King and Queen at Buckingham Palace, and the Prince and Princess of Wales at Marlborough House on Saturday, and in the afternoon the Queen of Würtemberg paid a visit to the Home of the Association of German Governesses, of which she is a patroness. She expressed herself as being much pleased with all the arrangements of the Home. Her Majesty left London on Monday night, other Royal personages leaving on Saturday.

### The War in the Far East.

IN an official report of the attack on Port Arthur by Admiral Togo, it is stated that the Japanese advance squadron attacked the Russian advance squadron, which was mostly outside the bay. The attack lasted 40 minutes and did considerable damage. The Japanese casualties were four killed and 54 wounded. Admiral Alexeieff, in command of the Russian fleet, reports that the torpedo transport Yenesei, was blown up by a submarine mine in the line of mine defences at Port Arthur on Thursday last week. The captain, three other officers, and 92 men were lost. All civilians and foreigners have been ordered to leave Port Arthur. So far as Japan is concerned, all war correspondents are detained in Tokio.

### Speech by the Japanese Emperor.

ON Saturday the Japanese Minister in London issued the following interesting communication:—The eleventh of this month being the 2,564th anniversary of the accession to the Throne of the Emperor Jimiri, first Emperor of Japan, a banquet was given by his Majesty at the Imperial Palace. His Majesty thus addressed his guests: "Upon the occasion of this memorable anniversary it gives me great pleasure to entertain the foreign representatives, ministers, officers, and distinguished princes. It is, indeed, with great regret that circumstances beyond our control, have compelled us to sever peaceful negotiations with a neighbouring Power. We are, however, pleased to say that our relations of friendship are daily increasing in cordiality with those Powers which are so worthily represented here, and we desire most earnestly to draw those relations still closer. We propose the health and happiness of the Sovereigns and rulers of these Powers."

### Retirement of Lord Roberts.

IT is announced that in consequence of the abolition of the office of Commander-in-Chief, Field-Marshal Lord Roberts has retired from the War Office, but that at the special request of the Prime Minister he has consented to place his services at the disposal of the Committee of Imperial Defence. General Sir N. G. Lyttelton becomes Chief of the General Staff, General C. W. H. Douglas Adjutant-General to the Forces, General H. C. O. Plumer Quartermaster-General to the Forces, and General Sir J. W. Murray Master-General of the Ordnance.

### The Duke of Norfolk's Marriage.

AFTER three postponements, the wedding of the Duke of Norfolk and the Honourable Gwendolen Mary Constable-Maxwell, elder daughter of Lord and Lady Herries, took place on Monday in the private chapel at Everingham Park, Lord Herries' residence, near York. As there is no other Roman Catholic church in the district, the chapel has been used as a public place of worship, and Miss Constable-Maxwell had acted as organist and choirmaster. As the Duke and his bride are cousins, the special sanction of the Pope had to be obtained before the marriage could be celebrated. The bride wore a dress of white Duchesse satin, draped with old family lace, and a veil given her by the Marchioness of Bute. The Court train was of silver-embroidered gauze, lined with cloth of silver. She had a tiara of real orange blossoms in her hair. There were ten bridesmaids who wore dresses of ivory satin crêpe de Chine, with quaint fichus of old lace and scarves of red chiffon, the younger having sashes instead of scarves, and short frocks instead of long dresses. All wore white beaver hats trimmed with red chiffon and white plumes. Part of the honeymoon is being spent at Garrowby Hall, and the Duke and Duchess of Norfolk are expected at Arundel Castle early in March.

### New Play at the New Theatre.

THE latest production at the New Theatre is cleverly adapted from the French of M. Alfred Capus by Mr. Comyns Carr, under the title of "My Lady of Rosedale." The heroine, Mrs. Fitzallen, is obliged to sell her house, Rosedale, to support herself and her child, her husband—from whom she hopes shortly to be divorced—having run through all his wife's fortune. She finds it is so heavily mortgaged that unless she can obtain a very good price she will have practically nothing left. Ralph Wigram, a wealthy, elderly bachelor, comes forward as a purchaser, and knowing from experience what it is to be "hard up," and being sorry for Mrs. Fitzallen, to whom he is much attracted, he insists on giving twice the market price. This greatly annoys Lady Prothero, who hoped to have secured the millionaire for her stepdaughter, but all goes well until the husband hears of the good fortune which has come to Mrs. Fitzallen, when he returns, bullies his wife, and vows he will not go on with the divorce. A strong scene then ensues between the two men. Ultimately, Mr. Wigram persuades the husband to depart again, and when the curtain descends there is a prospect that ere long Mrs. Fitzallen will become Mrs. Wigram, and once more "My Lady of Rosedale." Sir Charles Wyndham admirably catches the spirit of the humorous, kind-hearted millionaire, and Miss Mabel Terry-Lewis is earnest and refined as the ill-used wife. Miss Mary Moore only takes the very small part of Lady Mordaunt, but acts it delightfully.

### Notes and Queries.

**FOR REGULATIONS SEE PAGE 277.**

*Lady Doctor.*

(182) I want to be a doctor, but do not know what steps I must take. Is there any book that will guide me?—*Querist.*

"The Englishwoman's Year-book" will give you most of the information you require. You could obtain further particulars by writing direct to the medical schools mentioned as being open to ladies.

*Dandruff.*

(183) *Sister G.* would be glad if the Editor would give her a cure for dandruff. Several things have been tried, but in vain.

Consult a medical man.

*Prison.*

(184) Will you kindly tell me where to apply for work in a prison reformatory, or infirmary, for a few months? I am a maternity nurse, and have done district nursing, but I want to gain a wider experience amongst the lower classes.—*Nurse S.*

Apply to Dr. Donkin, Prison Commissioner, Home Office, Whitehall, W.C.

*Maternity.*

(185) A confinement takes place 13 days before the date of engagement, consequently the nurse is unable to attend. Is she entitled to her fees?—*Nurse Rose.*

The nurse would of course take the case as soon as she was free, and would charge full fees from that date. If she loses the case entirely, it is usual for her to receive half fees.

*Certificate.*

(186) Can any one tell me of a course of study by post through which a certificate for nursing could be obtained? Owing to family difficulties I was unable to complete my term at the hospital, and consequently I have no certificate to show. I have had many years' experience.—*Inquirer.*

No course of study by post would qualify you for a nursing certificate.

*Hospital Training.*

(187) I am 19 years of age, will you kindly tell me if I am too young to enter a general hospital as probationer?—*L. E. B.*

Yes. You might, however, enter a children's hospital.

*Home.*

(188) Would you kindly tell me of a home where a lady, 68 years old, could be received? She is recovering from a severe illness, and requires more attention than her own people can give her. They would, of course, contribute to her support.—*A. G.*

The case seems suitable for the Woodside Home, Whetstone, N. Terms from £7 to £22 per quarter.

*Alexandra Nurses.*

(189) I shall be glad if you will give me information respecting the "Alexandra Nurses."—*Nurse Annie.*

Apply Soldiers and Sailors Families' Association, 23 Queen Anne's Gate, Westminster, S.W.

*Home.*

(190) Can you kindly tell me if there is a home under the Holloway Trusts where a lady mentally afflicted can be received and where part of the treatment is gardening and other useful occupations?—*A. S. B.*

The Secretary of the Holloway Sanatorium Hospital for the Insane, St. Ann's Heath, Virginia Water, will answer all questions.

*British Pharmacopœia.*

(191) Will anyone tell me where I can buy a second-hand "British Pharmacopiœa" in good condition?—*Nurse.*

Such books are generally to be had from second-hand booksellers in the vicinity of medical schools.

**Important Nursing Textbooks.**

"The Nursing Profession: How and where to Train." 2s. net; 2s. 4d. post free.

"A Handbook for Nurses." By Dr. J. K. Watson. 5s. net; 5s. 4d. post free.

"Practical Guide to Surgical Bandaging and Dressings." By Wm. Johnson Smith, F.R.C.S. 2s. post free.

"The Nurses' Dictionary of Medical Terms and Nursing Treatment." By Honnor Morten. 2s. post free.

"The Human Body: its Personal Hygiene and Practical Physiology." By B. P. Colton. 5s. post free.

"Art of Feeding the Invalid." (Popular Edition). 1s. 6d. post free.

"On Preparation for Operation in Private Houses. By Stanhope Bishop, F.R.C.S. 6d. post free

### For Reading to the Sick.

"LEAD THOU ME ON."

Lead, kindly Light, amid the encircling gloom,
Lead Thou me on;
The night is dark, and I am far from home,
Lead Thou me on.
Keep Thou my feet; I do not ask to see
The distant scene; one step enough for me.

I was not ever thus, nor prayed that Thou
Shouldst lead me on;
I loved to choose and see my path; but now
Lead Thou me on.
I loved the garish day, and, spite of fears,
Pride ruled my will: remember not past years.

So long Thy power hath blest me, sure it still
Will lead me on,
O'er moor and fen, o'er crag and torrent, till
The night is gone;
And with the morn those Angel faces smile,
Which I have loved long since, and lost awhile. Amen.

*J. H. Newman.*

All the dark events of our lives, those strange circuitous leadings which were so different to what we expected and had planned, all had their "mysteries," each had reference to "the things of God;" and hereafter every dark and heavy cross which by God's grace we were enabled to bear for Christ's sake, shall shine with undying lustre when its reverse side is displayed to our astonished sight. "The harvest truly is plenteous;" let us lay up our store, if blindly, yet gladly, knowing that "He is faithful that promised" "He abideth faithful;" He will ever be a covenant God to all who truly seek Him. Our Father plans for us, painfully it may often be, but grandly, on the mighty framework of eternal love. The more we try to live in the "spirit of glory," which rests on those who suffer for Christ, the higher will our daily life be; and at times we shall see, even in our saddest hours, a glimpse of that glory, reflected perchance in the brightened faith of another sufferer, who has learnt how God's grace is all-sufficient from watching its mighty power in our life.

*Mrs. C. Campbell.*

Blessed are we if, when all earthly things come at last to be ended, when the trials of life are drawing to a close, when the supreme moment is approaching, and the shadows of death are deepening around us, blessed, if we have not chosen the interests, the pleasures of a world fading into the dim distance, but instead . . . "the things which are above"—the treasures of eternity—"where Christ sitteth on the right hand of God.—*W. J. Knox Little.*

Well I know thy trouble
O My servant true;
Thou art very weary,
I was weary too;
But that toil shall make thee
One day all My own,
And the end of sorrow
Shall be near My Throne.

## Figures and Tables

**Figure f1:**